# Prediction Equation for Calculating Fat Mass in Young Indian Adults

**DOI:** 10.5812/asjsm.34862

**Published:** 2010-06

**Authors:** Jaspal Singh Sandhu, Giniya Gupta, Shweta Shenoy

**Affiliations:** Department of Sports Medicine and Physiotherapy, Guru Nanak Dev University Amritsar, India

**Keywords:** Body Mass Index, Young Adult, Skinfold thickness, Weights, Body Height

## Abstract

**Purpose:**

Accurate measurement or prediction of fat mass is useful in physiology, nutrition and clinical medicine. Most predictive equations currently used to assess percentage of body fat or fat mass, using simple anthropometric measurements were derived from people in western societies and they may not be appropriate for individuals with other genotypic and phenotypic characteristics. We developed equations to predict fat mass from anthropometric measurements in young Indian adults.

**Methods:**

Fat mass was measured in 60 females and 58 males, aged 20 to 29 yrs by using hydrostatic weighing and by simultaneous measurement of residual lung volume. Anthropometric measure included weight (kg), height (m) and 4 skinfold thickness [STs (mm)]. Sex specific linear regression model was developed with fat mass as the dependent variable and all anthropometric measures as independent variables.

**Results:**

The prediction equation obtained for fat mass (kg) for males was 8.46+0.32 (weight) − 15.16 (height) + 9.54 (log of sum of 4 STs) (R2= 0. 53, SEE=3.42 kg) and − 20.22 + 0.33 (weight) + 3.44 (height) + 7.66 (log of sum of 4 STs) (R2=0.72, SEE=3.01kg) for females.

**Conclusion:**

A new prediction equation for the measurement of fat mass was derived and internally validated in young Indian adults using simple anthropometric measurements.

## INTRODUCTION

Obesity is an important risk factor for diabetes mellitus, hypertension, hyperlipidemia and cardiovascular diseases^[1]^, and is a strong predictor of morbidity and mortality^[1, 2]^. Obesity as described by American College of Sports Medicine (ACSM, 2006) is described as an excessive amount of adipose tissue, which is defined in young adults as body fat>25% in males and >32% in females. According to World Health Organizations (WHO), there were about 1.6 billion overweight adults, aged 15 years and above and at least 400 million adults, who were obese worldwide in 2005.Obesity is a serious public health problem that is growing in countries with low or middle income^[3]^. Positive energy balance resulting in being overweight and obesity is usually calculated by weight and height indices, of which body mass index (BMI in kg/m^2^ is the most widely used. However BMI does not differentiate between fat and lean mass tissue^[4, 5]^. At a given BMI, Asians have significantly higher body fat content than whites and black do^[6]^. Measurement of fat mass is needed for more accurate assessment of overweight and obesity^[7]^. Many techniques have been developed to assess body composition in humans. The gold standard of body composition in a two compartmental model has been under-water weighing. It measures body density, from which fat and lean mass content are estimated, by assuming standard figures for density of these components^[8]^. Other robust methods include, total body potassium method^[9]^, total body water method^[10]^ and multicompartmental models of body composition^[11]^. However, for routine clinical and epidemiological approach, simpler and cheaper anthropometric measurements have been used to predict body composition in relation to body density by under-water weighing^[12]^. Equations, to predict body fat from anthropometric measurements allows the estimation of body composition without complex and costly techniques, and can be used easily for on field assessment. Most currently used predictive equations were derived from measurements of persons in affluent, industrialized western population of developed countries and they may be inappropriate for persons in underdeveloped or developing countries, who have different genotypic and phenotypic characteristics^[7]^. For example the equation proposed by Durnin and Womersley^[13]^, which was developed using under-water weighing method, overestimates body fat and% body fat in population of developing countries^[14]^. With increasing obesity at a younger age^[15]^ and reports of the metabolic syndrome becoming evident even in younger age^[16]^, we believe, it is relevant to accurately estimate fat mass in young Indian adults. Hence, the present study was conducted to derive an equation for estimating fat mass (determined by under-water weighing), based on simple anthropometric measurements such as weight, height and skinfold thicknesses (ST) (biceps, triceps, subscapularis and suprailliac).

## METHODS AND SUBJECTS

*Subjects:* The study population comprised of 118 apparently healthy subjects (male N=58;female N=60) aged (20–29 yrs). Written informed consent was obtained from all subjects. The Institutional Ethics Committee, Faculty of Sports Medicine and Physiotherapy, Guru Nanak Dev University, approved the study protocol. The study was conducted in the months of August–September, 2009.

*Anthropometric measurements:* Subjects were weighed in minimal clothing, using a digital load cell balance, (Soehnle, West Germany), which has a precision of 0.1 kg. The height of the subjects was recorded, without footwear using a vertically mobile scale (Holtain, Crymych UK) and expressed to nearest 0.1 cm. Body mass index (BMI) was calculated from height and weight as follows; BMI=weight (kg)/height^2^ (m).

Skinfold measurements were carried out in triplicate, in the standing position and the mean was taken for further calculation: biceps, triceps, subscapularis and suprailliac. The biceps skinfold thickness was measured as the thickness of a vertical fold raised on the anterior aspect of the arm, the triceps skinflold thickness was measured over the triceps muscle at a point midway between the lateral projection of the acromion process of scapula and the inferior margin of the olecranon process of ulna.The subscapular skinfold was picked up on a diagonal, inclined inferolaterally approximately 45^c^ to the horizontal plane in the natural cleavage line of the skin. The site is just inferior to the inferior angle of the scapula. The suprailiac skinfold was measured in the midaxillary line following the natural cleavage of skin. It is aligned inferomedially at 45^c^ to the horizontal plane. All measurements were standardized. The skinfold measurements were carried out to the nearest 0.2 mm using skinfold calipers (Holtain, Crymych and UK). The logarithm(log) of the sum of the four skinfolds was used during linear regression because the same was used in age and gender specific equations for calculating body density, from which estimates of percentage of body fat were made.

Hydrostatic under-water weighing: Body fat percentage was measured using the hydrostatic under-water weighing machine” Vacumed Turbofit 5.10” (www.vacumed.com). The subjects were directed to slowly expel the inhaled air prior to submerging and continue until complete exhalation. They were urged to move slowly into the tank to reduce the dynamic effect of possibly moving water. The total body was submerged and no part of the body was allowed to touch the bottom or the sides of the tank. The under-water weight was entered automatically in the computer when the standstill on the indicator lighted up. An average of three readings was taken as the final reading. The software estimated the residual lung volume using the following equations:

Male residual lung volume=Vital capacity×0.24

Female residual lung volume=Vital capacity×0.28

Final %body fat was automatically calculated by the software using Brozek's formula^[24]^.

*Statistical analysis:* The entire original data set (males N=58; females N=60) was randomly divided into 2 sets of data. One set comprising about half of data (males N=29; females N=30) was used to develop prediction equation (prediction set), while remaining (males N=29; females N=30) was used to validate the data (validation set). Linear regression was made out in the prediction set (males N=29; females N=30) between fat mass estimated from under-water weighing and height, weight and log of sum of skinfold thickness (biceps, triceps, subscapularis and suprailliac). Age was not included into this analysis because of the narrow age range of present group of subjects. The difference in fat mass obtained by the prediction equation was compared against the body fat obtained by under-water weighing method^[17]^.

## RESULTS

The mean age of entire group, (males N=58; females N=60) was 23.08±2.18 yrs. Difference was significant for weight, height, biceps, triceps and suprailiac skinfold thickness and non significant for fat mass, BMI, and subscapular skinfoldthickness between males and females (Table 1).

**Table 1:** Mean values of variables for differences based on sex

Variable	Males N=58	Females N=60	P value
**Weight (kg)**	65.09±9.57	54.4±10.77	<0.001
**Height (m)**	1.7±0.09	1.6±0.06	<0.001
**Fat mass (kg)**	16.73±4.84	15.64±5.51	0.2
**Biceps (mm)**	4.19 ±2.19	7.56±3.87	<0.001
**Triceps (mm)**	6.96±3.13	13.6±4.93	<0.001
**Subscapular (mm)**	9.05±3.67	11.96±5.44	0.06
**Suprailliac (mm)**	7.73±3.58	12.02±5.65	<0.001
**BMI (Kg/m^2^)**	22.35±2.3	22.31±4.1	0.9

Values are mean±standard deviation

The characteristics of subjects in the prediction and validation data sets were compared for both males and females. The difference was significant for weight and height and non significant for fat mass and log (sum of 4 skinfold thickness) in males, whereas in females difference was not significant for any of variables (Table 2). The prediction equation obtained for calculating fat mass in males was:

$$\begin{array}{l} \text{Fatmass}=8.46+0.317(\text{weight})-15.161(\text{height})+9.536(\text{log of sum of }4\text{STs})\\ \left({\text{R}}^{2}=0.53,\text{SEE}=3.42\text{kg}\right) \end{array}$$

**Table 2:** Mean values of variables for differences between prediction set and validation set

Variable	Male			Female		
	Prediction set N=29	Validation set N=29	P. value	Prediction set N=30	Validation set N=30	P. value
**Weight (kg)**	67.94± 4.81	62.24±9.56	0.02	54.06± 8.256	62.24±9.56	0.8
**Height (m)**	1.74± 0.07	1.67±0.09	0.01	1.56±0.06	1.56±0.06	0.9
**Fat mass (kg)**	16.72± 4.81	16.74±4.96	0.991	15.67±4.53	15.6±6.41	0.9
**Log (sum of 4 ST)**	1.42± 0.7	1.42±0.15	0.99	1.66±1.47	1.58±0.018	0.1

Values are mean±standard deviation

While the equation derived for females was:

$$\begin{array}{l} \text{Fatmass}=-20.22+0.33(\text{weight})+15.161(\text{height})+9.536(\text{log of sum of }4\text{STs})\\ \left({\text{R}}^{2}=0.71,\text{SEE}=3.01\text{kg}\right) \end{array}$$

For the whole group of males (N=58), mean fat mass estimated by hydrostatic under−water weighing was 16.73±4.84 kg, while it was 16.76±3.52 kg when calculated from the derived equation. Paired t test showed no significant difference between the estimates of body fat mass from both methods. The mean difference for fat mass from under-water weighing was, −0.03±3.33 kg.

When similar statistical analysis was done for the whole group of females (N=60), mean fat mass estimated by hydrostatic under-water weighing was 15.64±5.51 kg while it was, 15.63±4.66 kg when calculated from the derived equation. Paired t test showed no significant difference between the estimates of body fat mass from both methods (*P*<0.05). The mean difference for fat mass from under-water weighing was 0.09±2.88kg.

In validation set of males (N=29), mean fat mass estimated by hydrostatic under-water weighing and by using derived equations was found to be 16.74±4.96 kg and 16.40±3.11kg respectively. No significant difference was found between both methods using paired t test.

In validation set of males (N=30), mean fat mass estimated by hydrostatic under-water weighing and by using derived equations was found to be, 15.60±6.41 kg and 15.44±5.5 kg, respectively. No significant difference was found between both the methods using paired t test.

**Fig. 1:**
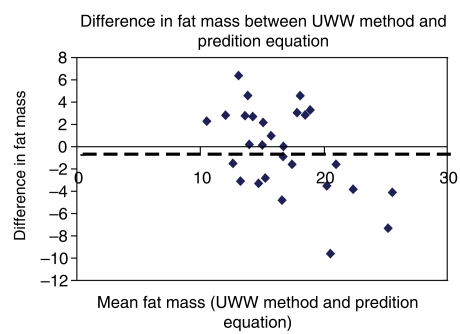
Dotted line represents mean difference in males (−0.33 kg)

**Fig. 2:**
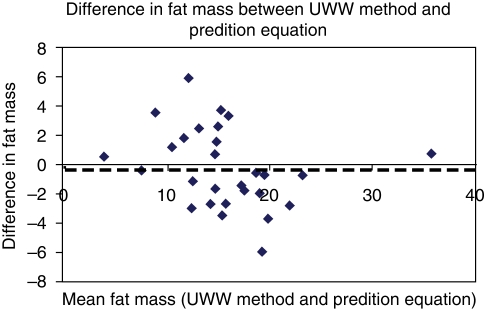
Dotted line represents mean difference in females (−0.16 kg)

The body fat calculated from the derived equation was compared to the estimates of body fat from under-water weighing by measuring the difference between them in the validation set of males (Fig. 1) and females (Fig. 2). The mean difference for fat mass from the derived equation was −0.3 kg for males and –0.16 kg for females. Paired t test analysis showed no significant differences in the estimate of fat mass between both the methods, in males as well as females.

## DISCUSSION

Higher amount of body fat and obesity are associated with increased risk of adverse health events and greater mortality^[1]^. Simple clinical measures of growth such as height and weight are unable to provide precise information with regard to this fact. Therefore, body composition assessments are used to meet these requirements in clinical medicine and research^[18]^. Many simple anthropometric techniques such as ST, BMI and other equations based on height and weight are preferred to assess body composition, in order to provide adequate information that is required in clinical and research practice, especially in low budget and field settings. Research in the past has been undertaken to test the reliability of the prediction equations based upon various anthropometric parameters in different populations. Since body composition varies with ethnicity^[11]^, equations that have been derived in western population are often not suited for Indian population^[19]^. There are a variety of ST equations, which are used, e.g. Sloan (1967), Durnin and Womersley (1974), Jackson and Pollock (1978) and etc., but Durnin and Womersley's (4 site equation) is the most commonly used and appears to be best among the poor bunch^[13, 20]^. Despite its lack of precision and specificity in estimating body composition at an individual level, this equation is commonly used for on field assessment of body composition because it is simple and easy to perform^[21]^. Because of the different fat distribution and age related changes of body fat, as compared to the western population, the equation cannot be used on Indians^[22, 23]^. Thus the present study aimed to derive a prediction equation for the measurement of fat mass in young Indian males and females based on simple anthropometric measurements.

The fat mass estimated using the derived equation, did not show significant difference from the fat mass calculated by using hydrostatic under-water weighing, in the validation set, as well as whole group of males and females.

Bland and Altman plot provided additional information, regarding the underestimation of fat mass using the derived equation (−0.3 kg for males and −0.16 kg for females). A prediction equation's R^2^ value is certainly important; however it should not be considered the only criterion when choosing a proper equation. In our derived equation, R^2^ was 0.53 and 0.72 for males and females respectively.

One of the various techniques available for estimating body fat is under-water weighing. With this method it has been found that in children and adolescents, body density increases with age and body fat decreases with age^[12]^. These findings point out that, we must use prediction equations specific for each age group. In our study we chose a narrow age range of 20–29 years for both males and females, thus our derived equation is specific for the age group of young Indian adults.

The hydrostatic under-water weighing machine that we used in our study is based on a two compartmental model (fat and fat free mass) which assumes that, when calculating total body density, the relative amounts and densities of bone, muscle and water, comprising the fat free mass, are essentially the same for all individuals, regardless of age, gender and race or fitness level^[24]^. It is now known that this is not the case. It would have been ideal to use four compartmental models as a criterion method with a higher degree of precision, but it was not possible in the present study because of unavailability of adequate resources.

In this study we chose subjects with a wide range of BMI 16.00–39.3 kg/m^2^ (females), 17.89–29.41 kg/m^2^ (males), as we wanted to derive an equation that worked across a wide range.

While the problem of undernutrition still exists in many parts of India, the additional burden of obesity due to increasing sedentary life style and junk food habits in the urban and economically sound areas is really alarming. Highest percentage of obese women is found in Punjab (29.9%)^[26]^. We derived and internally validated a prediction equation for young adults in the Punjabi Indian population. We believe that accurately estimating body fat percentage (which has a high correlation with the risk of developing heart diseases, metabolic syndrome and diabetes) in this young population would help to focus attention on the option of a healthier lifestyle e.g. increasing physical activity and low fat diet.

Indian Population is polygentic and is an amazing amalgamation of various races and cultures.On the basis of physical anthropology Indian population can be classified into various groups^[25]^. One of the limitations of present study is that, most of the subjects included were from Punjabi population.So, the validation of derived regression equation in all the Indian groups is questionable and needs to be investigated in further studies.

## CONCLUSION

The present study documented a prediction equation for the measurement of fat mass in young Indian adults (males and females), which used the hydrostatic under-water weighing method and is based on simple anthropometric measurements. We recommend testing the equation's accuracy and precision in various Indian groups (and races) to fully understand its strength and weakness.
